# Darier disease: the use of dermoscopy in monitoring acitretin treatment^☆^

**DOI:** 10.1016/j.abd.2021.05.021

**Published:** 2022-07-16

**Authors:** Catalina Silva-Hirschberg, Raúl Cabrera, María Paz Rollán, Alex Castro

**Affiliations:** a Department of Dermatology, Facultad de Medicina, Clínica Alemana, Universidad del Desarrollo, Santiago, Chile; b Department of Pathology, Facultad de Medicina, Clínica Alemana, Universidad del Desarrollo, Santiago, Chile

**Keywords:** Acitretin, Darier disease, Dermoscopy, Retinoids, Therapy

## Abstract

Darier disease is an uncommon autosomal dominant inherited disease, caused by a mutation in the *ATP2A2* gene. The clinical findings are hyperkeratotic papules on the trunk, scalp, face, and neck, maceration of intertriginous areas, palmar pits, whitish papules on the oral mucosa and nail abnormalities. The main histopathologic findings are acantholysis and dyskeratotic keratinocytes. Dermatoscopic features are comedo-like openings with a central polygonal yellowish/brownish structure, surrounded by a whitish halo. First-line treatment includes acitretin. Five reports have been published describing Darier disease dermatoscopic findings. Herein, we report for the first time a patient under acitretin treatment and dermatoscopic follow-up.

## Case report

**Figure 1:**
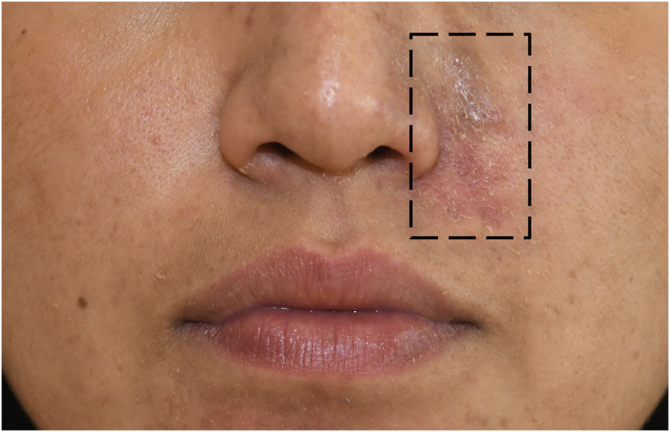
Clinical findings before acitretin treatment.

**Figure 2:**
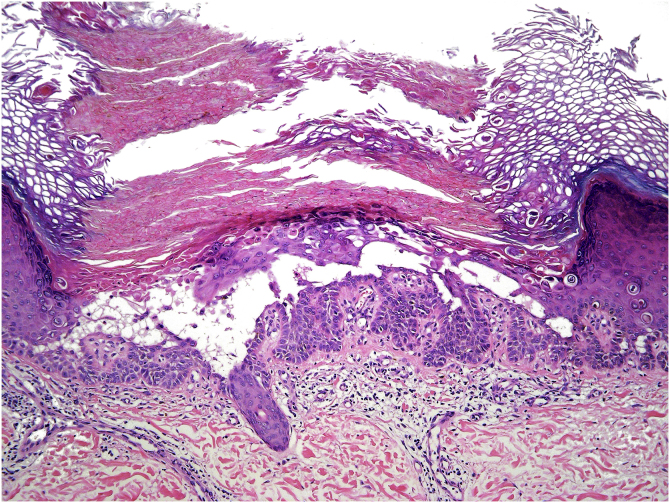
Light microscopy shows hyperkeratosis, suprabasal clefting, and dyskeratotic cells. There is a mild lymphocytic infiltrate in the papillary dermis (Hematoxylin & eosin, ×100).

**Figure 3:**
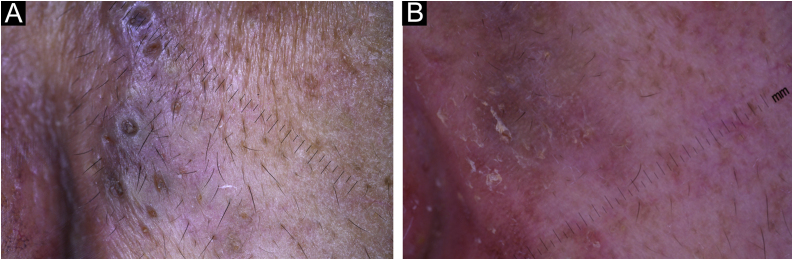
Dermatoscopic examination (×10). (A), Before acitretin treatment. Big comedo-like openings (300 to 500 microns) with a central hyperkeratotic structure, surrounded by a whitish scaling halo. (B), After acitretin treatment. Few white shiny clods with a pinkish homogeneous background.

**Figure 4:**
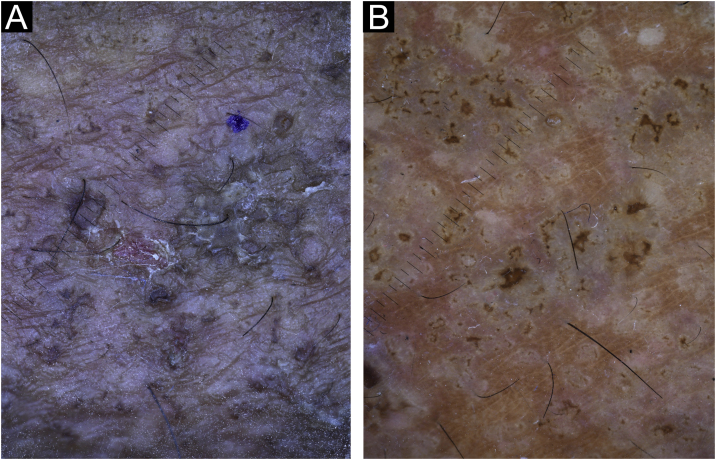
Dermatoscopic examination (×10). (A), Before acitretin treatment, multiple confluent big comedo-like openings with a central hyperkeratotic structure surrounded by a whitish scaling halo are seen in the legs. (B), After acitretin treatment, white shiny clods (of different size) and crust are found in the skin.

A 33-year-old woman presented since childhood history of persistent scaly papules on both legs and face. Physical examination revealed multiple small keratotic, crusted, brown-to dark-brown papules located in the left nasolabial fold of the face (Fig. 1) and the extensor surface of both legs. The nails and oral mucosa were not affected. Under dermoscopy (DermLite DL4, 10×; 3 Gen, San Juan Capistrano, CA, USA) oval-shaped yellow-brownish big comedo-like areas were found, surrounded by a white halo in a pinkish background. Skin biopsy was compatible with Darier disease (Fig. 2). The patient was treated with acitretin 25 mg daily and after six months of treatment a remarkable clinical and dermatoscopic improvement was achieved in the skin lesions of the face (Fig. 3) and a good response in the legs (Fig. 4).

## Discussion

Darier disease (DD) or Darier-White disease is an autosomal dominant inherited keratinization disorder.^1^ It has complete penetrance but variable expression with a negative impact on life quality. First described by Jean Darier and James Clarke White in 1889.^1^, ^2^ It is caused by a mutation in the ATP2A2 gene ‒ in all cases ‒ which encodes the sarco/endoplasmic reticulum Ca2+ATPase type 2 (SERCA2).^2^ The prevalence ranges from 1/30,000 to 1/50,000, and affects both genders in equal proportion, without ethnic differences.^1^

The classical clinical findings are hyperkeratotic papules in a seborrheic distribution involving the trunk, scalp, face, and lateral aspects of the neck. They are usually symmetric lesions, but mosaic Darier disease can occur if lesions follow Blaschko's lines unilaterally or if they are distributed in focal areas of increased severity superimposed on a background of generalized DD.^3^ The latter corresponds to the present case, where unilateral facial lesions were found. Other features include maceration of intertriginous areas, palmar pits, and whitish papules in the oral mucosa (hard palate). Nail abnormalities can be found showing; V‑shaped notches at the distal edge of the nail, longitudinal white and red alternating streaks, subungual hyperkeratosis, splinter hemorrhages, and nail fragility.^2^, ^4^

The main histopathologic findings are acantholysis due to loss of cell adhesion and dyskeratotic keratinocytes (premature cells). These dyskeratotic cells are described as “corp ronds” in the spinous-granular layer and “grains” in the stratum corneum.^1^

Dermatoscopic features have also been described in DD. They include comedo-like openings with a central polygonal or star-like yellowish/brownish structure, surrounded by a whitish halo.^4^, ^5^ A pink homogeneous structureless background is associated with white scales and dotted or linear vessels.^5^ First-line treatment in DD includes topical or systemic retinoids, becoming acitretin a valuable alternative since 1988.^6^ Only five reports have been published describing DD dermatoscopic findings.^4^, ^5^, ^7^, ^8^ In this case, the authors have been able to compare the results of acitretin treatment ‒ before and after ‒ using dermatoscopy.

Acitretin is the active metabolite of etretinate. It was introduced in 1988 and replaced etretinate, mostly due to a better pharmacokinetic profile and lesser adverse effects.^9^ Acitretin strongly binds to cellular retinoic acid-binding protein, which transports retinoic acid from the cytosol to the nucleus. It modifies the transcription of over 500 genes, reduces proliferation, and increases keratinocyte differentiation. It inhibits induction of T-helper-17 (Th-17) cells by suppressing Interleukin-6 (IL-6) and promotes differentiation of T regulatory cells. Acitretin reduces keratinocyte production of vascular endothelial growth factors and inhibits neutrophil migration.^10^ In summary, it modulates keratinocytes proliferation and differentiation and has immunomodulatory and anti-inflammatory activities.^9^ Therefore, acitretin has been shown to have a consistent therapeutic efficacy in disorders of keratinization (e.g., bullous ichtyosiform erythroderma, lamellar ichtyosis, pityriasis rubra pilaris).^9^ It has become the first choice for the treatment of Darier Disease.^6^

In the present study’s case, the effect of acitretin was remarkable but the most important aspect was to demonstrate the effectiveness of this drug using dermatoscopic follow-up. Before treatment, the patient had big comedo-like openings (measuring 300 to 500 microns as is shown in the dermatoscope scale) with a central hyperkeratotic yellowish/brownish structure, surrounded by a whitish halo (Fig. 3A). After 6-months of treatment these lesions involuted almost completely, leaving a few white shiny structures that could only be seen under dermoscopy as a result of the inflammatory and fibrotic process that followed the treatment with acitretin (Fig. 3B, Fig. 4B).

We confirmed the importance of dermatoscopy as a useful diagnostic tool for clinical diagnosis in dermatology. In this case, dermatoscopy was also essential for monitoring the treatment of the patient with DD.

## Financial support

None declared.

## Authors' contributions

Catalina Silva-Hirschberg: Approval of the final version of the manuscript; critical literature review; manuscript critical review; preparation and writing of the manuscript.

Raúl Cabrera: Approval of the final version of the manuscript; intellectual participation in propaedeutic and/or therapeutic management of studied cases; manuscript critical review; preparation and writing of the manuscript.

María Paz Rollán: Approval of the final version of the manuscript; critical literature review; manuscript critical review.

Alex Castro: Approval of the final version of the manuscript; intellectual participation in propaedeutic and/or therapeutic management of studied cases; manuscript critical review.

## Conflicts of interest

None declared.
